# Respiratory enterovirus D68: virology, clinical surveillance, host-pathogen interactions, and therapeutic prospects

**DOI:** 10.3389/fcimb.2026.1774711

**Published:** 2026-03-05

**Authors:** Yanshan Gui, Bing Han, Jialong Wei, Danlei Sun, Laixian Zhou, Shuchen Yuan, Wenzhan Xie, Hui Feng

**Affiliations:** 1 School of Medicine, Chongqing University, Chongqing, China; 2 Department of Laboratory Medicine, The Affiliated People’s Hospital of Chongqing University, Chongqing, China; 3 Department of Laboratory Medicine, The First Affiliated Hospital of Chengdu Medical College, Sichuan, China

**Keywords:** antivirals, clinical surveillance, escape, immune response, neurotropic enterovirus

## Abstract

Enterovirus D68 (EV-D68), a unique enterovirus resembling human rhinoviruses, was long considered to cause only sporadic outbreaks of mild, self-limiting respiratory infections mainly in children. However, over the past decade, EV-D68 has exhibited a biennial outbreak pattern across multiple regions worldwide, coinciding with an increased incidence of severe respiratory illnesses and cases of acute flaccid myelitis (AFM) in children. The immune system plays a crucial role in providing rapid and effective defense. Nonetheless, our knowledge of the complex interactions between EV-D68 and the host immune responses is still very limited. Additionally, clinical detection of EV-D68 remains challenging, and there are no FDA-approved vaccines or antiviral treatments available. Therefore, ongoing research should focus on understanding the pathogenic mechanisms of EV-D68, as well as the development of reliable diagnostic methods and therapeutic options to control EV-D68 spread. This review intends to examine the initiatives undertaken for clinical surveillance of EV-D68 outbreaks, the immune responses elicited by EV-D68, and its strategies for immune evasion. Additionally, it explores recent advancements in antiviral drug development, thereby providing a comprehensive overview of current knowledge and identifying prospective directions for future research.

## 1 Introduction

Enterovirus D68 (EV-D68, also known as EV68) is a small non-enveloped, single-stranded, positive-sense (+) RNA virus within the genus Enterovirus of the Picornaviridae family. For several decades, EV-D68 was infrequently detected throughout surveillance efforts and was primarily associated with mild respiratory illnesses. However, in 2014, the United States experienced a nationwide outbreak of EV-D68 that was linked not only to severe respiratory illnesses but also to cases of acute flaccid myelitis (AFM), a disabling, polio-like neurologic disorder mainly affecting young children under the age of five (Pastula et al., 2014; Division of Viral Diseases et al., 2015; Greninger et al., 2015; Dábilla et al., 2025). Since then, accumulating evidence has documented an increasing incidence of clinically severe cases attributed to EV-D68 infection worldwide, including Canada (Skowronski et al., 2015; Fall et al., 2022), China (Zhang et al., 2015; Wang et al., 2020; Tang et al., 2021), West Africa (Fall et al., 2020), and Europe (Pfeiffer et al., 2015; Ruggieri et al., 2017; Karelehto et al., 2019; Andres et al., 2022; Peltola et al., 2023). Although the prevalence of EV-D68 infections markedly declined during the COVID-19 pandemic, a notable re-emergence of EV-D68 cases was reported in Europe between 2021 and 2022 (Benschop et al., 2021). In the Lombardy region of northern Italy, EV-D68 constituted the predominant enterovirus type from mid-August 2024 onward (Pariani et al., 2024). Moreover, EV-D68 has been incorporated into the World Health Organization viral priority pathogens list 2024 (Ukoaka et al., 2024).

Despite the recent public health threat posed by EV-D68, advancements in outbreak surveillance and elucidation of its pathogenesis have been eclipsed by the prioritization of poliovirus (the most well-known enterovirus) and other enteroviruses. Notably, EV-D68 is distinctive among enteroviruses due to its unique biological characteristics and clinical symptoms that resemble those of human rhinoviruses, the most common causes of viral respiratory infections (Oberste et al., 2004; Cassidy et al., 2018). Furthermore, EV-D68 infections tend to occur predominantly during or immediately following the summer-fall season (Baker et al., 2024), complicating clinical diagnosis and surveillance efforts. Nevertheless, consistent with other enteroviruses, the pathogenesis and clinical outcomes of EV-D68 infection are determined largely by a complex interplay between host immune responses and viral factors, as elaborated below. Currently, no effective vaccines or specific antiviral treatments exist for the prevention or clinical management of EV-D68 infection. Accordingly, it is essential to enhance global efforts for more accurate monitoring and management of EV-D68 circulation, while integrating recent findings that elucidate its intricate interactions with host immune systems. In this context, we will examine the molecular biology of EV-D68, discuss practical detection techniques and surveillance networks to track EV-D68 outbreaks, and summarize current understanding of its complex interplays with host immune responses. Additionally, we will outline potential therapeutic strategies targeting the virus.

## 2 Molecular biology and life cycle of EV-D68

**Figure 1:**
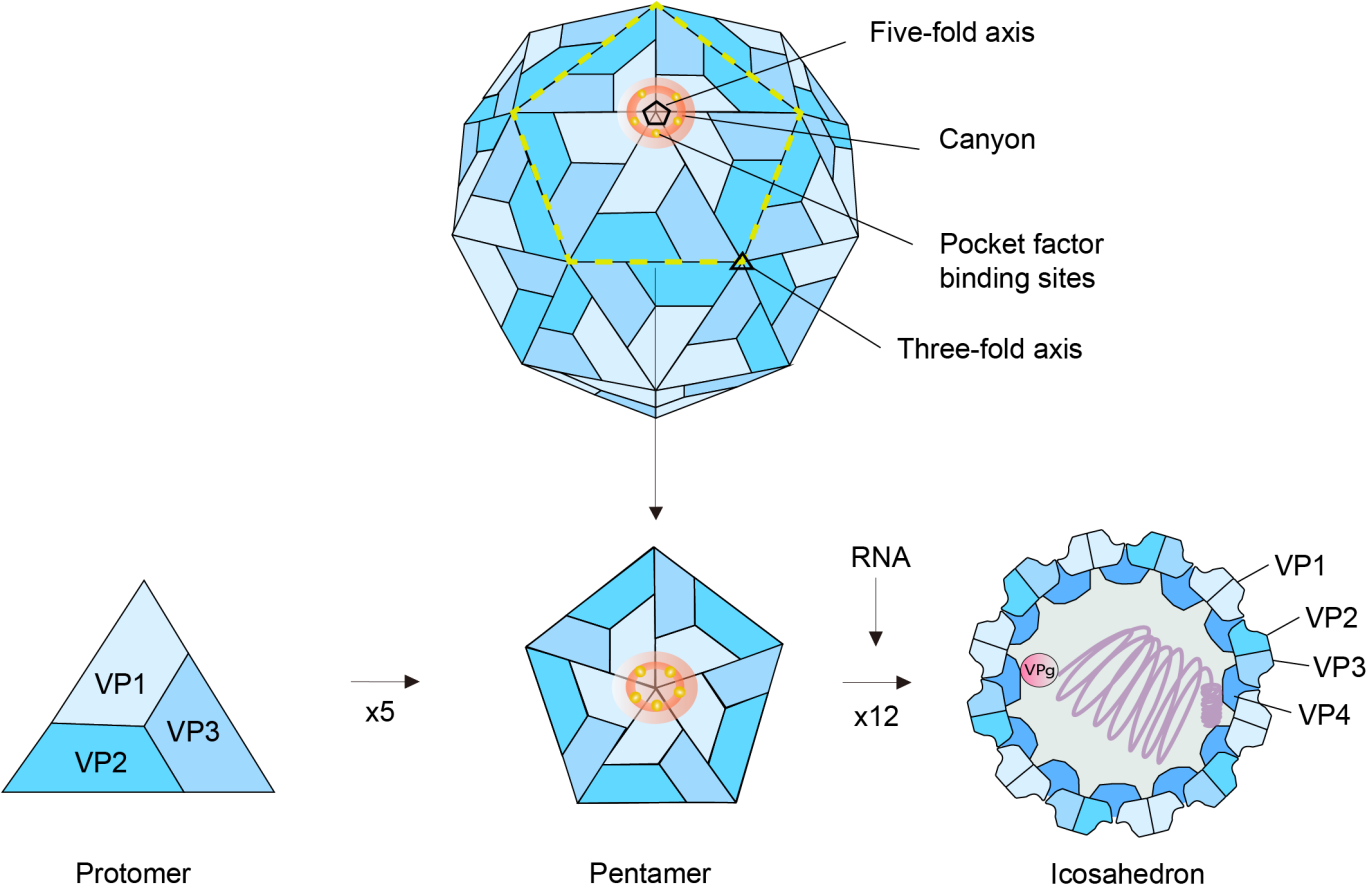
Diagrammatic representation of EV-D68 architecture. The virion of EV-D68 consists of an icosahedral capsid shell surrounding a single strand of a positive-sense RNA genome, with a diameter around 30 nm. Its capsid is composed of 60 protomers, each comprising four structural proteins designated VP1 through VP4. Five protomers assemble to form a pentamer, and twelve such pentamers then form the icosahedral shell, concomitantly packaging the viral RNA genome. Within mature virions, VP4 is located internally, whereas VP1, VP2, and VP3 comprise the external surface of the particle. At the capsid vertices, these latter proteins converge to establish alternating three-fold and five-fold symmetry axes. Near the five-fold axis vertices, a conserved structural feature termed the “canyon” is present. At the base of this canyon, each VP1 subunit contains a hydrophobic pocket that accommodates a host-derived lipid-like molecule, commonly referred to as the “pocket factor.” VPg, virus encoded protein.

**Figure 2:**
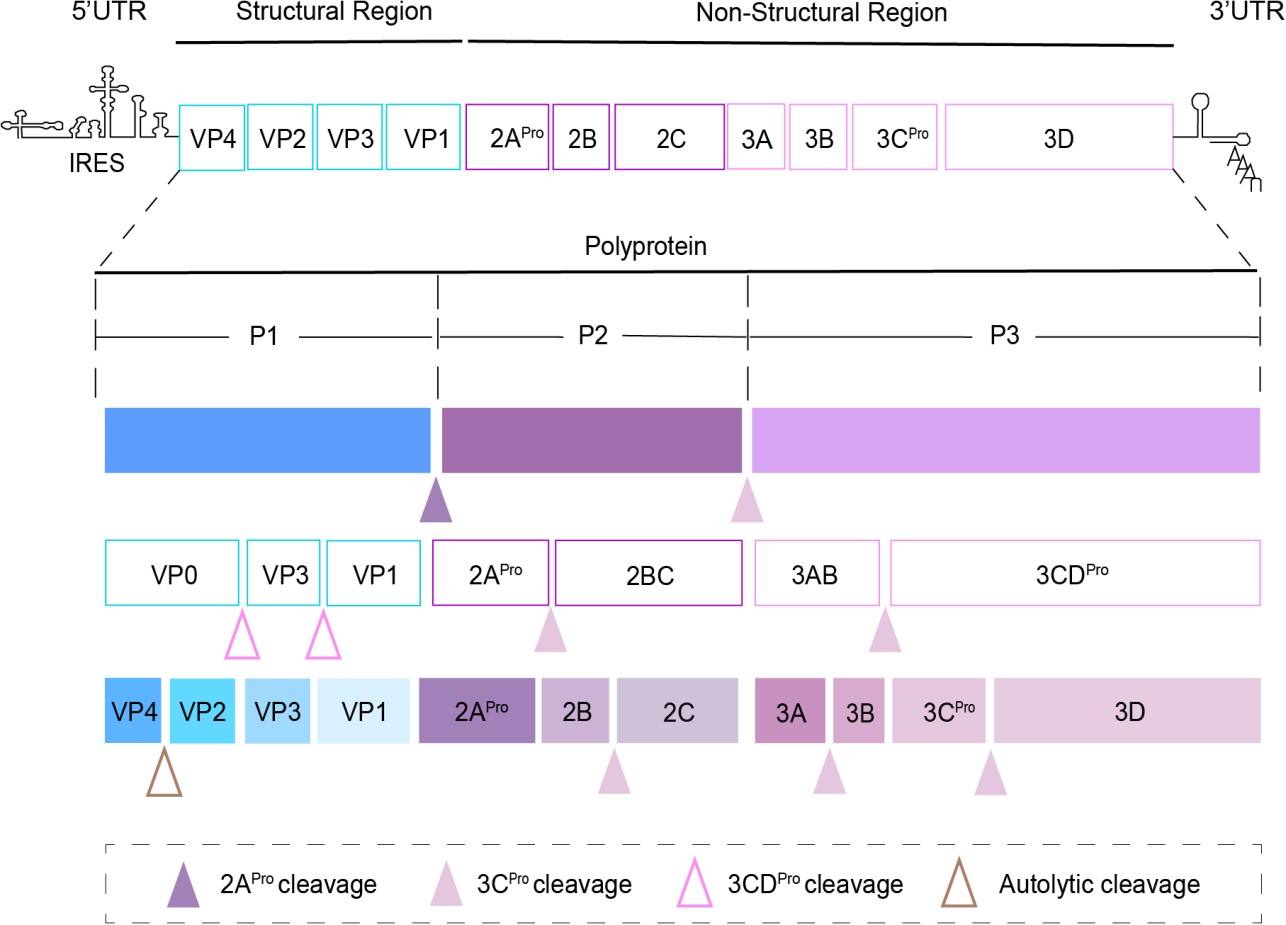
Schematic illustration of the enterovirus genome and polyprotein processing. The genome of EV-D68 is a 7.4 kb single stranded positive-sense RNA. It comprises a single open reading frame (ORF) flanked by 5’ and 3’ untranslated regions (UTRs), which contain an internal ribosome entry site (IRES) and a polyadenylate tail, respectively. The polyprotein encoded within the enterovirus genome undergoes sequential proteolytic cleavage mediated by the viral proteases 2A^pro^ and 3C^pro^, leading to the production of 4 mature structural proteins (VP1-VP4) and 7 mature non-structural proteins (2A-2C, 3A-3D).

The capsid of EV-D68 virions is comprised of 60 copies of each of the four structural proteins (VP1-VP4), which are organized into subunits in a pseudo T = 3 symmetry (**Figure 1**). In this arrangement, VP1, VP2, and VP3 are oriented outwardly, while VP4 is located on the interior surface of the capsid. Moreover, the VP1 proteins converge at the vertices to form pentameric assemblies characterized by fivefold symmetry, which are encircled by a canyon-like structure. Notably, EV-D68 particles possess canyons that are narrower, shallower and discontinuous compared to those in other enteroviruses (Liu et al., 2015b; Baggen et al., 2018). Although the biological importance of such unique canyons remains to be elucidated, they may account for the markedly reduced stability of EV-D68 under acidic conditions and elevated temperatures relative to other enteroviruses, a trait similarly observed in other picornaviruses (McKnight and Lemon, 2018). Besides capsid, the (+) RNA genome of EV-D68 contains an open reading frame flanked by terminal untranslated regions (UTRs) (**Figure 2**). This genome acts directly as messenger RNA for the translation of a single precursor polyprotein, a common feature shared by all picornaviruses.

**Figure 3:**
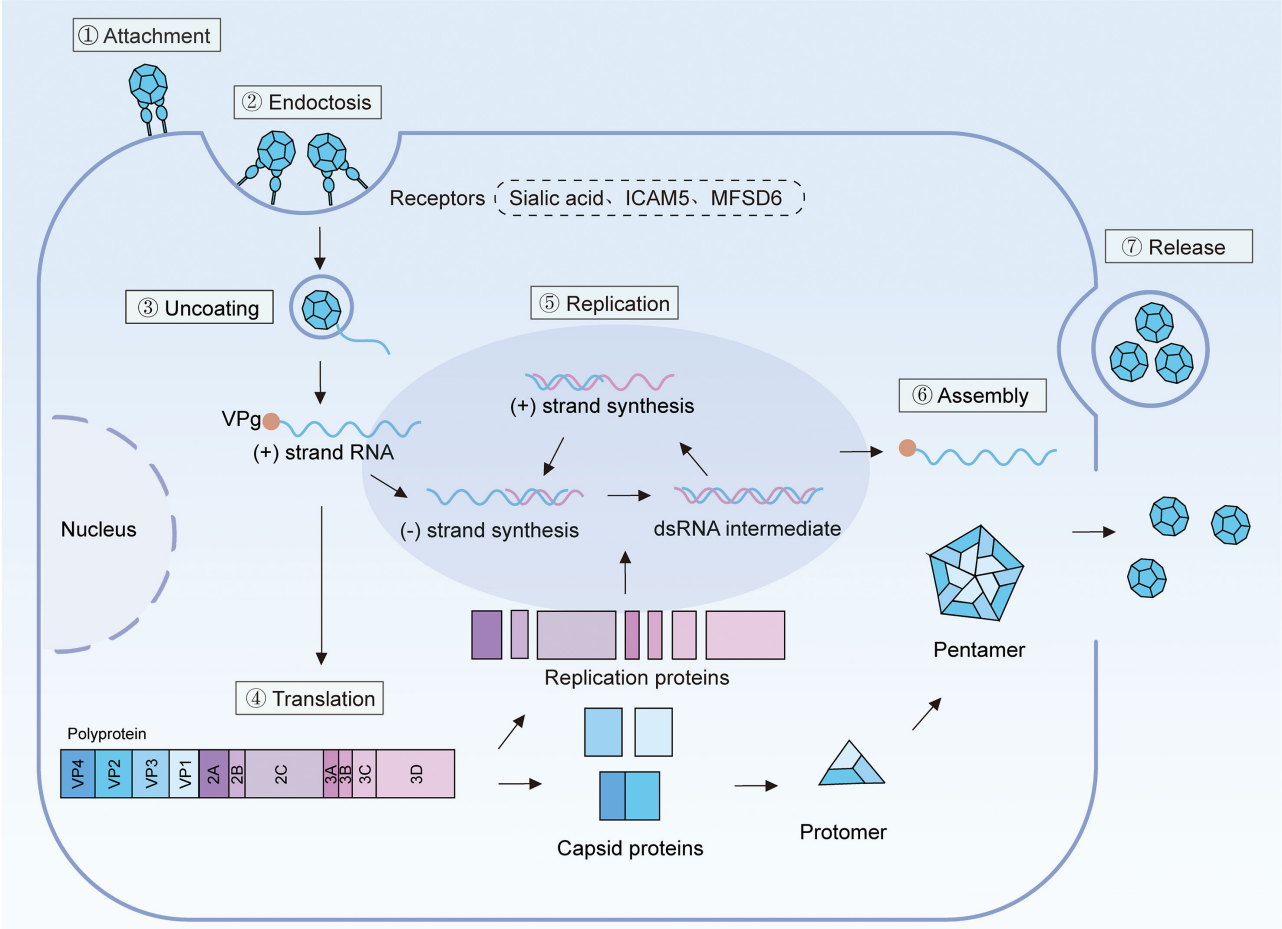
Depiction of the life cycle of EV-D68. EV-D68 initiates infection by attaching to the host cell membrane through interactions with specific receptors, including MFSD6, ICAM5, and sialic acid, facilitating its internalization via endocytosis (steps ①-②). Subsequently, the viral particle releases its genomic RNA into the cytoplasm through pores formed in the endosomal membrane (step ③). The viral RNA undergoes translation followed by co-translational proteolytic cleavage, producing eleven principal proteins. These include structural proteins VP1 through VP4, which are essential for capsid assembly, and non-structural proteins 2A through 3D, which are involved in viral RNA replication (step ④). Genome replication occurs on specialized membranous replication organelles, beginning with the synthesis of a negative-strand (−) RNA intermediate. This (−) RNA serves as a template for the production of new positive-strand (+) RNA genomes by the viral RNA-dependent RNA polymerase (3D) (step ⑤). Finally, the newly synthesized viral RNA and capsid proteins are assembled into mature virions, which are subsequently released from the host cell either via extracellular vesicles or through direct cell lysis (step ⑥). ICAM5, intercellular adhesion molecule 5; MFSD6, major facilitator superfamily domain-containing protein 6.

EV-D68 life cycle begins with binding to cell surface receptors (**Figure 3**). The first receptor proposed for EV-D68 was sialic acid (Uncapher et al., 1991). Indeed, multiple EV-D68 strains, including the prototype Fermon strain and contemporary isolates from 2010 to 2011, exhibited markedly reduced binding to the cell surface following sialic acid removal (Uncapher et al., 1991; Liu et al., 2015a). Moreover, genes implicated in the biosynthesis, transport, and conjugation of sialic acid were shown to be essential for EV-D68 infection (Baggen et al., 2016). Notably, while both α2,6- and α2,3-linked sialic acids can function as cellular EV-D68 receptors, the virus displays a preferential affinity for α2,6-linked sialic acids. Interestingly, however, certain EV-D68 strains isolated from 2012 onward, including three strains from the 2014 outbreak in the United States, have demonstrated the capacity to infect cells independently of sialic acid (Baggen et al., 2016; Hixon et al., 2019). This observation suggests an evolutionary adaptation of EV-D68 to exploit multiple cellular receptors, which may account for its sudden outbreaks and potential neurotropic characteristics (Brown et al., 2018). Consistent with this notion, the neuron-specific intercellular adhesion molecule 5 (ICAM5) has been identified as a cellular receptor for both sialic acid-dependent and -independent EV-D68 strains across various cell types (Wei et al., 2016). However, given the absence of readily detectable ICAM5 expression in the human respiratory tract in vivo, it is plausible that additional receptor(s) exist. Indeed, two independent laboratories have recently reported that the host membrane transporter major facilitator superfamily-domain-containing protein 6 (MFSD6) serves as an entry receptor for respiratory EV-D68 in both cell lines and primary respiratory and neural cells (Liu et al., 2025b; Varanese et al., 2025). The binding of EV-D68 virions to their receptors is followed by uncoating. This process enables viral genome delivery into the cytoplasm via a pore formed in the endosomal membrane, and is reliant on the host phospholipase PLA2G16 (Staring et al., 2017). Although this mechanism has not been explicitly validated for EV-D68, a dual glycan receptor-binding EV-D68 strain isolated during the 2012 outbreak in the Netherlands has been shown to utilize a strategy that evades PLA2G16 dependency (Baggen et al., 2019).

Once delivered into the cytosol, the viral RNA is translated into a single, large polyprotein. This polyprotein undergoes sequential proteolytic cleavage mediated by viral proteases including 2Apro, 3Cpro and 3CDpro, resulting in the production of viral structural proteins (VP0, VP1 and VP3), viral replication proteins (2A–2C, 3A–3D), as well as several functional cleavage intermediates (e.g. VP0 and 3CDpro) (Baggen et al., 2018; Elrick et al., 2021). It is noteworthy that, unlike hepatitis A virus—a member of the Picornaviridae family—which predominantly generates the 3ABCpro cleavage intermediate, enteroviruses preferentially cleave at the junction between 3B and 3Cpro (Feng et al., 2019; Sun et al., 2023). Viral genome replication occurs within virus-induced membranous structures known as replication organelles, and is mediated by the RNA-dependent RNA polymerase 3D. This process begins with the synthesis of a negative-strand copy of the incoming viral genome, forming a double-stranded RNA replication intermediate. The negative strand subsequently serves as a template for the synthesis of new positive-strand RNAs. These newly synthesized viral RNAs are then packaged into nascent virions (Galitska et al., 2023). Although EV-D68 has traditionally been characterized as obligate lytic viruses released upon cell rupture, emerging evidence indicates that it can also exit host cells nonlytically in extracellular vesicle structures (Jassey et al., 2023, 2024a).

## 3 Clinical surveillance of EV-D68 infections

Molecular diagnostic techniques targeting viral RNA have been widely utilized for confirming EV-D68 infection. For instance, sequencing the entire or partial VP1 capsid protein gene via the conventional Sanger method has long been considered the gold standard for enterovirus typing, and has been widely applied worldwide (Nix et al., 2006; Bubba et al., 2020). Besides VP1, sequencing analyses targeting other genomic regions, particularly the VP4/VP2 capsid protein genes, have become integral to the diagnosis and surveillance of enteroviruses (Shaw et al., 2014; Itani et al., 2023). In addition to sequencing, several reverse transcriptase-polymerase chain reaction (RT-PCR) assays have been developed for detecting EV-D68 infection (Piralla et al., 2015; Wylie et al., 2015; Zhuge et al., 2015; Ikuse et al., 2021; Howson-Wells et al., 2022). Furthermore, nasopharyngeal specimens positive for EV-D68 have been demonstrated to be suitable for quantitative RT-PCR pooled testing to identify local virus infections (Spradley et al., 2025). Although these methods offer a rapid way for testing EV-D68, the virus undergoes continuous evolution during sustained circulation, particularly within the VP1 region (Hodcroft et al., 2022; Li et al., 2024a; Hirvonen et al., 2025). Recently, a droplet digital PCR (ddPCR) assay has been established (Grizer et al., 2024). This assay targets conserved regions within the VP1 gene, enabling the detection of a broad range of EV-D68 strains, including the historical Fermon prototype and contemporary isolates circulating up to 2024. Therefore, ddPCR may exhibit reduced susceptibility to sensitivity loss caused by primer-target mismatches.

Owing to the rapid evolution of enteroviruses, the detection of an enterovirus in a patient does not necessarily confirm an epidemiological link to an outbreak (Sanjuan, 2012; Itani et al., 2023). Consequently, beyond the technical scope of clinical diagnosis, coordinated surveillance networks is crucial for elucidating the epidemiology and controlling the spread of EV-D68. For example, the European Non-Polio Enterovirus Network implements standardized cross-border surveillance, which proved instrumental in tracking the resurgence and genotypic evolution of EV-D68 during the 2021–2022 outbreak (Harvala et al., 2018; Simoes et al., 2024; Hirvonen et al., 2025). In the United States, the Centers for Disease Control and Prevention has established a multi-system surveillance framework. Within this framework, the National Enterovirus Surveillance System integrates typing data from 11 laboratories, revealing that EV-D68 constituted 55.9% of enterovirus-positive specimens between 2014 and 2016 (Abedi et al., 2018). Concurrently, the New Vaccine Surveillance Network—an active surveillance platform encompassing seven medical centers—has systematically delineated the epidemiological profile of EV-D68 and established its association with AFM (Kujawski et al., 2019; Shah et al., 2021). Beyond Europe and North America, the Asia-Pacific Network for Enterovirus Surveillance has supported unified pathogen identification and data sharing for enteroviruses including EV-D68 by facilitating collaboration among academic and clinical institutions in Cambodia, Malaysia, Vietnam, and Taiwan, and China (Chiu et al., 2020).

## 4 Host immune responses and EV-D68 counteractions

Until now, the molecular mechanisms underlying its pathogenesis remain poorly understood. Nevertheless, like all other viruses, the interplay between host immunity and viral evasion strategies is crucial for the pathogenesis of EV-D68 infection.

### 4.1 Innate immunity

**Figure 4:**
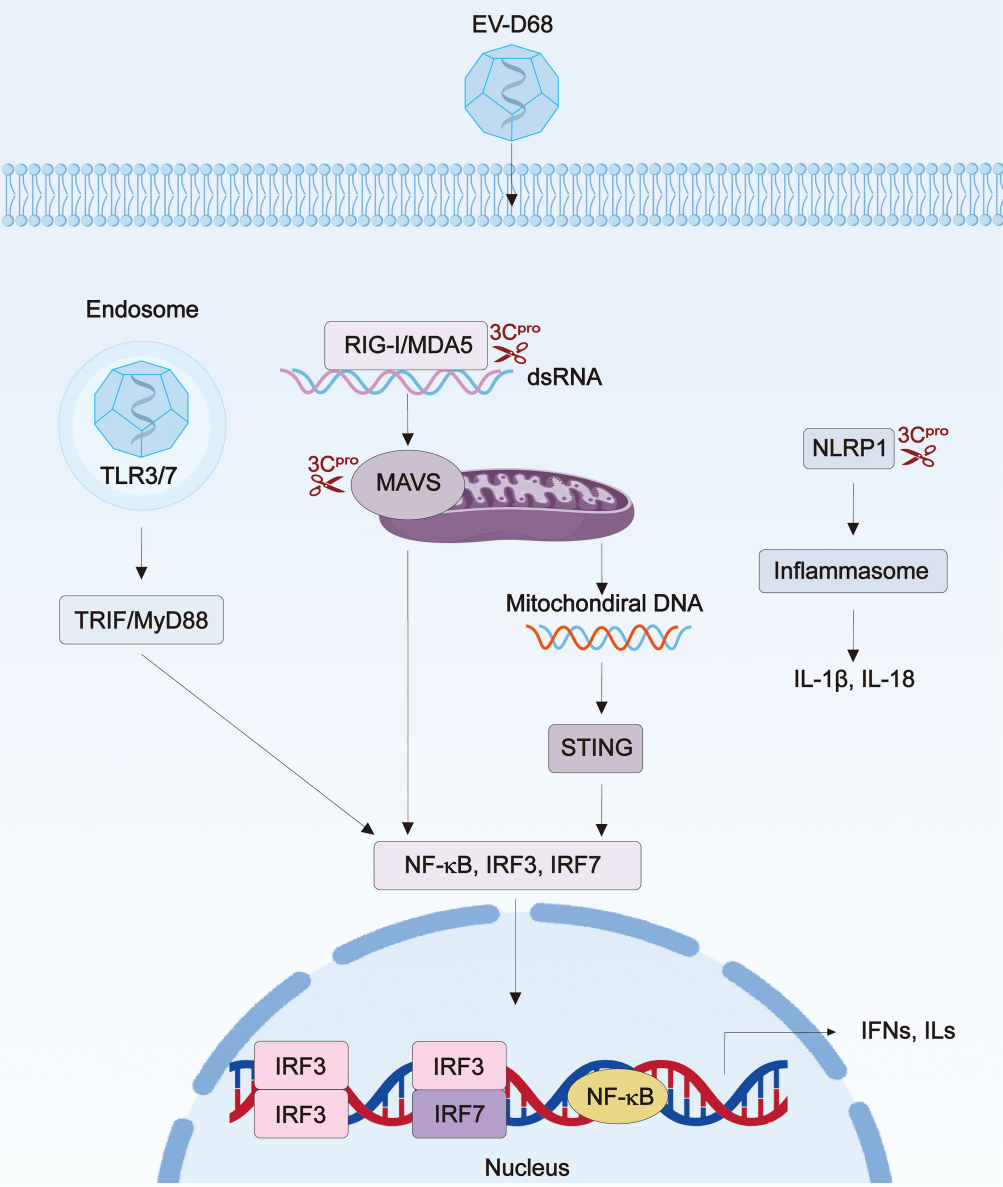
Interactions between EV-D68 and the host innate immune responses. Upon EV-D68 infection, toll-like receptors (TLR), such as TLR3 and TLR7, along with retinoic acid-inducible gene I-like receptors (RLRs), including RIG-I and MDA5, recognize viral RNA or its replicative intermediates. This facilitates the recruitment of downstream adaptors proteins, namely MyD88/TRIF for TLRs and MAVS for RLRs. Subsequently, these adaptor proteins initiate the nuclear translocation of transcription factors IRF3/7 and NF-κB, leading to the induction of interferons (IFNs) and interleukins (ILs). Besides, EV-D68 infection induces mitochondrial damage, releasing mitochondrial DNA into the cytoplasm and activating STING-mediated antiviral signaling. Additionally, EV-D68 3C^pro^ cleaves host NLRP1 at its N-terminus, thereby releasing the C-terminal fragment to initiate inflammasome activation. To promote EV-D68 propagation in host cells, viral factors such as 2A^pro^, 3C^pro^ and VP3, are known to target TLR, RLR, STING pathways to counteract antiviral innate immunity. RIG-I, retinoic acid-inducible gene I; MDA5, melanoma differentiation-associated protein 5; MyD88, myeloid differentiation primary response 88; TRIF, toll/interleukin-1 receptor domain-containing adaptor-protein-inducing interferon-β; MAVS, mitochondrial antiviral signaling protein; NLRP1, NOD-like receptor family pyrin domain containing 1.

EV-D68 encounters the epithelial cells lining the mucosal surfaces of the respiratory system during transmission. Notably, while EV-D68 can infect individuals across all age groups, children comprise the majority of reported cases and outbreaks (Simoes et al., 2024; Fall et al., 2025). These findings suggest that the mucosal innate immunity serves as the principal protective mechanism against EV-D68 infection (**Figure 4**).

The innate immune system generally initiates antiviral responses through pattern recognition receptors (PRRs), such as Toll-like receptors (TLRs) and retinoic acid-inducible gene I (RIG-I)-like receptors (RLRs) (Feng et al., 2021). This process starts with the detection of pathogen-associated molecular patterns (PAMPs), including viral RNA genomes introduced into host cells and double-stranded RNA (dsRNA) produced during viral replication. Specifically, upon RNA recognition, TLRs recruit adaptor proteins such as myeloid differentiation primary-response protein 88 (MyD88) or Toll/interleukin (IL)-1 receptor domain-containing adaptor-protein-inducing interferon-β (TRIF), whereas RLRs recruit the mitochondrial antiviral signaling protein (MAVS) (Farooq et al., 2021; Liu et al., 2022). These interactions trigger downstream signaling cascades involving various kinases that subsequently activate nuclear factor kappa-B (NF-κB), IFN regulatory factors 3 and 7 (IRF3 and IRF7), resulting in the synthesis of interferons (IFNs) and pro-inflammatory cytokines (Yi et al., 2017). Indeed, respiratory infections with recent EV-D68 isolates have been associated with a marked induction of both type I and III IFNs, suggesting a critical role for innate immune responses in the acute control of viral infection (Freeman et al., 2021; Grizer et al., 2025). Consistent with this, it has been demonstrated that the expression of host TLR7 proteins is selectively upregulated following EV-D68 infection, and that Vesatolimod (GS-9620, a TLR7 agonist) broadly inhibits the replication of circulating EV-D68 isolates (Li et al., 2024b). Likewise, TLR3 signaling is believed to contribute to host defense against EV-D68 infection, as the virus counteracts TLR3-dependent antiviral responses by cleaving its downstream adaptor protein TRIF (Xiang et al., 2014). Besides TLRs, a recent study has revealed that EV-D68 induces type III IFN production via signaling mediated by melanoma differentiation-associated protein 5 (MDA5), a member of the RLR family (Chio et al., 2024). Beyond the involvement of TLRs and RLRs, the stimulator of interferon genes (STING), a crucial immune adaptor that mediates cyclic GMP-AMP synthase (cGAS) detection of cytosolic DNA fragments, also appears to participate in the host innate immunity to EV-D68 infection. This is supported by the evidence that the STING ligand cyclic AMP-IMP (cAIMP) significantly inhibits EV-D68 infection in cardiomyocytes (Garcia et al., 2023). Interestingly, this inhibitory effect is attributed to mitochondrial damage and the consequent release of mitochondrial DNA into the cytosol of infected cells, with tumor necrosis factor receptor-associated factor 3 (TRAF3) acting as a key mediator during STING activation (Zheng et al., 2023).

In addition to the well-established recognition of PAMPs, accumulating evidence underscores the significance of effector-triggered immunity (ETI) in human innate immune responses (Orzalli and Parameswaran, 2022; Castro and Daugherty, 2023; Remick et al., 2023). Notably, PRRs that assemble inflammasomes, such as caspase recruitment domain family member 8 (CARD8) and NLRP1, have recently been identified as innate immune sensors responsive to the enzymatic activities of diverse viral proteases (Wang et al., 2021; Planes et al., 2022). Interestingly, this phenomenon has also been documented in the context of human rhinovirus infection (Robinson et al., 2020). Mechanistically, the viral 3Cpro cleaves NLRP1 at the Q130^G131 site, a sequence that is conserved among enteroviruses (Fan et al., 2020; Tsu et al., 2021). Unlike other host proteins being targeted by viral proteases, this cleavage event induces proteasome-dependent degradation of the autoinhibitory N-terminal fragment of NLRP1, thereby releasing the C-terminal fragment to initiate inflammasome assembly (Feng et al., 2019; Robinson et al., 2020; Sun et al., 2023). Given the close similarity between rhinovirus and EV-D68, as well as the conserved nature of 3Cpro across picornaviruses, it is anticipated that EV-D68 3Cpro similarly activates NLRP1-dependent ETI. Consistent with this notion, EV-D68 3Cpro has been shown to cleave human NLRP1 and triggers inflammasome formation, albeit with a less robust response compared to that elicited by human rhinovirus (Tsu et al., 2021). Furthermore, a recent study has identified a single-nucleotide polymorphism in human CARD8 that confers the ability to sense rhinovirus 3Cpro activity, similarly implying the potential of EV-D68 infection in triggering CARD8-mediated ETI (Tsu et al., 2023).

### 4.2 Adaptive immunity

Besides innate immunity, adaptive immune responses contribute significantly to the defense against EV-D68 infection, primarily via humoral immunity and cellular immunity mechanisms (Duan et al., 2022). Indeed, a high prevalence of neutralizing antibodies targeting the EV-D68 prototype Fermon strain has been documented within the Finnish population, aligning with observations that EV-D68 can generate infectious progeny in leukocyte cell lines with monocytic, granulocytic, T cell, or B cell phenotypes (Smura et al., 2010). Similarly, humoral immune responses to EV-D68 have been demonstrated in patients with AFM (Mishra et al., 2019; Schubert et al., 2019). Moreover, both studies identified the VP1 capsid protein as the predominant epitope target, a pattern commonly observed in immune responses to other enteroviruses (Grifoni et al., 2019). Likewise, T cell-mediated immunity appears to play a crucial role in modulating AFM severity following EV-D68 infection. For instance, IL-17-dependent cytokine production supports a role for helper T cell responses in a mouse model of EV-D68 respiratory disease (Rajput et al., 2018; Yang et al., 2025). Consistent with this, significantly elevated nasopharyngeal IL-17 mRNA expression was detected in patients infected with EV-D68 during the 2014 epidemic period compared to those with rhinovirus infection (Rajput et al., 2018). Notably, an independent murine model of EV-D68 neurotropic disease similarly revealed increased T cell infiltration in spinal cord tissue relative to controls (Woods Acevedo et al., 2025). Moreover, flow cytometry analysis of spinal cord cells indicated that EV-D68 infection results in marked increases in both CD8+ and CD4+ T cell populations, while infiltration by neutrophils and monocytes remained minimal (Duan et al., 2025). It is noteworthy that the increased T cell infiltration was found to be associated with both CCR2, a receptor for several cytokines upregulated by EV-D68, and RAG1, a protein essential for lymphocyte maturation (Woods Acevedo et al., 2025). Therefore, it is highly possible that, similar to other enteroviruses, CD8⁺ T cell responses play a critical role in the control of EV-D68 infection (Woods Acevedo et al., 2025). Activated CD8⁺ T cells exert such effect by releasing cytokines such as IFN-γ and TNF-α, while concurrently enhancing the activity of peripheral immune cells (Xun et al., 2021; Liu et al., 2025a). Importantly, although these activated CD8⁺ T cells contribute to antiviral immune protection, their cytotoxic activity may also inflict damage on infected neurons. This immunopathological reaction is regarded as a key mechanism underlying the pathogenesis of AFM (Gonzalez-Dunia et al., 1997; Woods Acevedo et al., 2025).

### 4.3 Immune evasion strategies of EV-D68

While immune responses play a critical role in protection against EV-D68 infection, the virus also has the capacity to counteract immune activation. In particular, the viral proteases 2A^pro^ and 3C^pro^ have been extensively implicated as key factors in the suppression of these immune responses. For instance, the 3C^pro^ of EV-D68 prevents MAVS recruitment by binding to MDA5, thereby interfering with MDA5-mediated IFN signaling (Rui et al., 2017). This 3C^pro^ also downregulates the expression of RIG-I and tripartite motif-containing protein 25 (TRIM25, an E3 ubiquitin ligase facilitating RIG-I activation), and thus suppresses type I IFN responses (Xiao et al., 2021). Furthermore, 3C^pro^ directly cleaves host transforming growth factor β-activated kinase 1 (TAK1) and TRIF, leading to the inhibition of TLR-initiated activation of downstream transcription factors such as NF-κB and IRF3, as well as the suppression of type I IFN expression (Xiang et al., 2014; Rui et al., 2017). Additionally, 3C^pro^ mediates the cleavage of IRF7 and signal transducer and activator of transcription 1 (STAT1), and this cleavage abrogates their nuclear translocation, thereby inhibiting the expression of IFNs and IFN-stimulated genes (ISGs) (Xiang et al., 2016; Li et al., 2024c). Likewise, EV-D68 2A^pro^ has been lately shown to target host tumor necrosis factor receptor-associated factor 3 (TRAF3), subverting STING-dependent innate immune responses (Li et al., 2021; Zheng et al., 2023; Jassey et al., 2024a).

EV-D68 has also been demonstrated to employ both 2A^pro^ and 3C^pro^ to manipulate host cellular processes in a manner that favors viral replication and egress (Nouwen et al., 2025; Schipper et al., 2025). For example, typical stress granule proteins tend to bind to the 3’-UTR of EV-D68 genome RNA and thus inhibit viral replication (Cheng et al., 2020). Nonetheless, EV-D68 utilizes its 2A^pro^ to efficiently suppress the formation of stress granules (Visser et al., 2019). Although the underlying mechanism remains unclear, this action relies on the proteolytic activity of 2A^pro^. Beyond its proteolytic activity, EV-D68 2A^pro^ promotes viral RNA replication through a specific interaction with the cytosolic SETD3 protein in a manner independent of its well-known methylation activity (Diep et al., 2019). Moreover, a recent study has demonstrated that EV-D68 employs both 3C^pro^ and 2A^pro^ to cleave host proteins such as La-related protein 1 (LARP1) and poly(A)-binding protein cytoplasmic 1 (PABPC1), thereby redirecting host global translation machinery toward viral genomic RNA translation and replication (Tan et al., 2025). Interestingly, EV-D68 3C^pro^ and 2A^pro^ also differentially modulate members of the gasdermin family, inactivating the pyroptosis executioner gasdermin D (GSDMD) while simultaneously inducing GSDME-mediated cell pyroptosis, a phenomenon similarly observed during SARS-CoV-2 infection (Ma et al., 2021; Planes et al., 2022; Shen et al., 2024). Although the biological importance of this shift requires further investigation, it has been observed that GSDME-mediated pyroptosis triggered by the virus proceeds more slowly than canonical caspase-dependent pyroptotic cell death, thereby providing a temporal window conducive to virus replication (Shen et al., 2024). Additionally, both 2A^pro^ and 3C^pro^ have been shown to disrupt the composition of the nuclear pore complex (NPC) via directly cleaving a relatively small number of nucleoporins (Zinn et al., 2025). Notably, while this cleavage does not appear to affect RNA export, it impairs NPC barrier permeability and inhibits the nuclear-cytoplasmic transport of protein cargoes, consistent with the fact that viruses target the NPC to facilitate replication (De Jesús-González et al., 2021). Similarly, EV-D68 3C^pro^ targets and cleaves the host Cullin 3 protein, resulting in the suppression of E3 ubiquitin ligase activity. This process subsequently stabilizes the viral capsid protein VP1 by obstructing its degradation through the ubiquitin-proteasome system (Li et al., 2025b).

Besides the viral proteases, other EV-D68 proteins have been gradually recognized for their roles in evading host immune responses. Notably, the structural protein VP3 of EV-D68 has been shown to co-localize and interact with MAVS, specifically disrupting IRF7-dependent type I interferon signaling pathway (Chen et al., 2025). Moreover, VP3 suppresses IFN production mediated by IRF7 (Kang et al., 2023). Mechanistically, VP3 interacts with IRF7, thereby inhibiting virus-induced phosphorylation and nuclear translocation of IRF7, as well as competitively inhibiting TNF receptor associated factor 6 (TRAF6)-mediated ubiquitination of IRF7. In addition, EV-D68 3D protein interacts with phosphoglycerate mutase 5 (PGAM5) and promotes mitofusin 2-driven mitochondrial fusion in human epithelial cells, which in turn suppresses RIG-I-initiated type I IFN production (Yang et al., 2021).

## 5 Research on potential anti-EV-D68 drugs

Given the lack of specific antiviral agents, there is a great need for the development of therapeutic drugs to treat EV-D68 infections. In this section we summarize recent findings on antiviral inhibitors targeting the virus life cycle (**Table 1**).

**Table 1:** Potential anti-EV-D68 drugs and their therapeutic targets.

Type	Anti-EV-D68 drug	Target	Reference
Viral capsid inhibitors	Pleconaril	VP1 hydrophobic pocket	(Liu et al., 2015b)
	Pterostilbene	Viral capsid	(Chuang et al., 2025)
	R856932	VP1 hydrophobic pocket	(Ma et al., 2019)
	Vapendavir	VP3	(Sun et al., 2015)
	Avoenin	VP3	(Arita et al., 2020)
	MFSD6-Fc(CH3)	Viral capsid	(Liu et al., 2025b)
	15C5	icosahedral three-fold axes	(Zheng et al., 2019)
	11G1	icosahedral five-fold axes	(Zheng et al., 2019)
Viral polymerase inhibitors	NITD008	3D polymerase	(Yan et al., 2021)
	L-alanine	3D polymerase	(Yan et al., 2021)
	FNC	3D polymerase	(Xu et al., 2020)
	AcTU	3D polymerase	(Li et al., 2020; Liu et al., 2023)
Inhibitors of viral proteases	Telaprevir	2A^Pro^	(Musharrafieh et al., 2019; Tan et al., 2023)
	Rupintrivir	3C^pro^	(Witherell, 2000; Xie et al., 2020; Azzolino et al., 2024)
	V-7404	3C^pro^	(Rhoden et al., 2015; Kankam et al., 2021)
Viral components	Fluoxetine	2C	(Ulferts et al., 2013; Rhoden et al., 2015; Bauer et al., 2019; Manganaro et al., 2020; Kankam et al., 2021)
	Jun6504	2C	(Ulferts et al., 2013; Bauer et al., 2019; Manganaro et al., 2020; Li et al., 2025a)
	R523062	2C	(Ma et al., 2020b; Li et al., 2025a)
	A-967079	2C	(Ma et al., 2020b; Tan et al., 2024)
	Emetine	IRES	(Tang et al., 2020; Tan et al., 2024)
	Licochalcone A	IRES	(Chuang et al., 2024)
Host factors	DAS181	α2,6-linked sialic	(Rhoden et al., 2015)
	Enviroxime	PI4KIIIβ	(Sun et al., 2015; Smee et al., 2016)
	Itraconazole	OSBP	(Strating et al., 2015; Guedán et al., 2017)
	OSW-1	OSBP	(Strating et al., 2015; Guedán et al., 2017)
	CRT0066101	PKD	(Strating et al., 2015; Guedán et al., 2017)

### 5.1 Viral capsid inhibitors

Targeting the viral capsid shell represents a pivotal strategy for inhibiting viral infections. Capsid-targeting antiviral agents frequently bind to hydrophobic pockets on the capsid surface, thereby blocking virus entry and/or uncoating by modulating capsid rigidity and stability. For example, Liu et al. demonstrated that pleconaril, a capsid-binding compound previously shown to be active against rhinoviruses, binds to a hydrophobic drug-binding pocket in VP1 of the Fermon strain (Liu et al., 2015b). Similarly, pterostilbene, a natural compound found in blueberries and other plants, exhibits broad-spectrum inhibitory effects against EV-D68 during early stages of infection, including viral attachment, receptor binding, and enhancement of virion stability (Yang et al., 2023; Chuang et al., 2025). Molecular docking analyses have revealed a strong binding affinity between pterostilbene and a hydrophobic pocket within VP1. Additionally, the tetrazole compound R856932 has been identified as a novel capsid-binding inhibitor, demonstrating inhibitory activity against multiple contemporary EV-D68 strains with single-digit to submicromolar potency (Ma et al., 2019). Mechanistic investigations indicate that R856932 binds to the hydrophobic pocket of VP1, thereby preventing viral uncoating and subsequent release of the viral genome into the cytoplasm. Furthermore, recent virtual screening and rational design efforts have led to the identification of a series of novel quinoline analogues targeting VP1 as anti-EV-D68 agents (Li et al., 2022). Besides, a recent study reported that platelet factor 4 inhibits EV-D68 infection (Lv et al., 2025). Mechanistically, a 15-amino acid peptide (C15) located at the C-terminus of platelet factor 4 mediates its antiviral activity by specifically binding to a conserved domain (residues 155-170) within VP3, thereby disrupting viral attachment to host cell surfaces. Like these capsid binders, vapendavir has demonstrated broad-spectrum antiviral activity against various enteroviruses, including EV-D68 (Sun et al., 2015). 2R,4R-(12Z,15Z)-heneicosa-12,15-diene-1,2,4-triol, commonly referred to as avoenin and derived from avocado, was also identified as a potent inhibitor of EV-D68 (Arita et al., 2020). Moreover, the emergence of resistant EV-D68 mutations occur at specific amino acid residues within VP3, suggesting that the inhibitory effect of avoenin may result from its direct binding to this viral protein.

In recent years, neutralizing antibodies specific for EV-D68 have also been developed. For instance, two monoclonal antibodies, designated 15C5 and 11G1, have been shown to neutralize the virus and thus block viral attachment (Zheng et al., 2019). Interestingly, the atomic structures of EV-D68 in complex with these two antibodies revealed distinct mechanisms of neutralization. 15C5 binds to the viral capsid at the icosahedral three-fold axes and triggers the transformation of mature virions into classical uncoating intermediates (A-particles), thereby mimicking viral engagement with the functional ICAM5 receptor. In contrast, 11G1 specifically recognizes the A-particle form and engages the capsid loci at the icosahedral five-fold axes (Zheng et al., 2019). Recently, a promising human monoclonal antibody, EV68-228, was isolated from the blood of patients who had recovered from EV-D68 infection (Vogt et al., 2020). By binding near the fivefold symmetry axes of the virion particles, it shows robust neutralizing activity against a wide range of modern EV-D68 isolates. EV68–228 also confers protection to AG129 (deficient in receptors for interferon α/β and γ) adult mice against both respiratory and neurologic disease when administered either before or after infection. Additionally, EV68–228 produced in Nicotiana benthamiana (EV68-228-N) has demonstrated efficacy in arresting the progression of paralysis when delivered post-onset in an immunocompetent neonatal Swiss–Webster mouse model (Rudy et al., 2025). It has now advanced to a phase 1 randomized, double-blind, placebo-controlled clinical trial aimed at assessing its safety and tolerability in healthy adults (ClinicalTrials.gov ID: NCT06444048). Beyond these above antibodies, a recombinant protein complex comprising the MFSD6 ectodomain fused to an Fc fragment (MFSD6-Fc(CH3)) was developed successfully, effectively blocking EV-D68 infection *in vitro* and preventing lethality in neonatal mice (Liu et al., 2025b).

While targeting virus attachment and entry is efficient therapeutic strategy, it is important to acknowledge associated limitations. For example, pleconaril has demonstrated little efficacy against circulating EV-D68 strains at clinically relevant concentrations. Moreover, it did not receive approval from the Food and Drug Administration (FDA) due to concerns regarding its safety profile (Senior, 2002; Tyler, 2015). Furthermore, the virus has been observed to rapidly develop resistance to capsid-binding agents (De Palma et al., 2008; Lanko et al., 2021).

### 5.2 Viral polymerase inhibitors

Polymerase inhibitors are generally categorized into two main categories—nucleoside and non-nucleoside inhibitors—based on their structural characteristics and action mechanisms. Nucleoside inhibitors typically consist of nucleoside analogs that are mistakenly incorporated into the growing RNA strand by the viral polymerase, thereby terminating RNA chain elongation and inhibiting viral genome replication. In contrast, non-nucleoside inhibitors impede polymerase activity via various mechanisms, such as allosteric modulation or competitive binding at the catalytic site of the polymerase.

To date, several nucleoside inhibitor candidates have been identified that effectively suppress EV-D68 replication. For instance, the adenine analogue 2’-ethynyl-7-deoxyadenosine (NITD008) and its phosphoamidate derivatives demonstrate *in vitro* antiviral activity against EV-D68 (Yan et al., 2021). Among these, the derivative featuring a cyclohexyl ester of L-alanine (termly 15l) exhibited the most potent antiviral efficacy, characterized by a high selectivity index against both EV-D68 and EV-A71 at low concentrations, surpassing the activity of NITD008 (Yan et al., 2021). Additionally, the cytidine analog 2’-deoxy-2’-β-fluoro-4’-azidocytidine (commonly referred to as FNC or azvudine), a potent HIV inhibitor entered into clinical phase II trials in China, has shown broad-spectrum antiviral activity against various enterovirus genotypes, including EV-D68, both *in vivo* and in neonatal mouse models (Xu et al., 2020). Likewise, 4’-fluorouridine (4’-FlU) has been identified as a broad-spectrum anti-enterovirus agent with efficacy against EV-D68 (Chen et al., 2024).

The non-nucleoside inhibitors identified so far include acylthiourea-based 4-(tert-butyl)-N-((4-(4-(tert-butyl)benzamido)phenyl)carbamothioyl) benzamide (AcTU) and NADPH. Importantly, the crystal structure of EV-D68 3D polymerase in its apo state and in complex with NADPH demonstrated that NADPH occupies the RNA template binding channel of EV-D68 3D polymerase, a binding site that partially overlaps with that of AcTU (Li et al., 2020; Liu et al., 2023). Considering that the structure of EV-D68 polymerase closely resembles that of other enteroviral polymerases, it is reasonable to anticipate that inhibitors targeting the 3D polymerases of enteroviruses may also exhibit efficacy against EV-D68 (van der Linden et al., 2015; Wang et al., 2016, 2017). However, the potential for suboptimal antiviral activity remains a concern.

### 5.3 Inhibitors of viral proteases

Enteroviral proteases play a vital role in both the replication of viral genome RNA and the evasion of host immune defenses, making them among the most successfully validated targets for therapeutic intervention. For instance, telaprevir, an FDA-approved medication for the treatment of hepatitis C virus infections, has been identified as a potent inhibitor of EV-D68 2A^pro^ through a nearly irreversible, biphasic binding mechanism (Musharrafieh et al., 2019; Tan et al., 2023). Beyond showing submicromolar to low-micromolar efficacy against several modern circulating neurotropic strains of EV-D68 in various human cell lines, telaprevir also significantly improves paralysis outcomes in a murine model of EV-D68 associated AFM (Frost et al., 2023).

Similarly, rupintrivir (AG7088), an inhibitor that was originally developed for rhinovirus 3C^pro^ and has shown antiviral activity against the main protease (M^pro^) of SARS-CoV-2, has been confirmed to exhibit *in vitro* protective effects against clinical isolates from the three major EV-D68 clusters (Witherell, 2000; Xie et al., 2020; Azzolino et al., 2024). However, rupintrivir showed limited efficacy in murine models of EV-D68 respiratory and neurological infection (Hurst et al., 2019). This is not entirely unforeseen, considering that several compounds have been elucidated for inhibiting not only SARS-CoV-2 M^pro^ but also the 2A^pro^ and 3C^pro^ from EV-D68 (Ma et al., 2020a). In contrast, the inhibitor V-7404 has demonstrated potent activity against EV-D68 isolates and no easily notable adverse effects (Rhoden et al., 2015; Kankam et al., 2021). Like other antiviral agents, resistance development poses a challenge for enteroviral 3C^pro^ inhibitors, although resistance emerges more slowly compared to capsid inhibitors (Lanko et al., 2021). Nonetheless, recent research has provided valuable insights for the rational design of inhibitors with enhanced resistance profiles, facilitating the development of effective antiviral therapies for EV68 infections (Azzolino et al., 2025). Additionally, comprehensive computational screening has identified natural compounds, such as withaferin-A and baicalin, as promising antagonists of EV-D68 3C^pro^ (Alsahag, 2025). Despite their high binding affinities and interactions with key residues within the active site of 3C^pro^, the *in vivo* efficacy of these compounds remains to be demonstrated.

### 5.4 Inhibitors targeting other viral components

2C protein is among the most highly conserved viral proteins, and its characteristics render it a promising target for the development of antiviral agents (Li and Zhang, 2022). The FDA-approved antidepressant fluoxetine and its analogues have demonstrated effective inhibition of EV-D68 replication *in vitro* (Ulferts et al., 2013; Bauer et al., 2019; Manganaro et al., 2020). However, fluoxetine has neither ameliorated motor impairments nor reduced viral loads in a mouse model of EV-D68-associated AFM (Hixon et al., 2017), and clinical trials have similarly failed to show therapeutic benefit in AFM patients (Messacar et al., 2019). Despite these observations, its potential as a treatment for chronic enterovirus encephalitis was noted in a patient unresponsive to conventional high-dose intravenous immunoglobulin therapy (Gofshteyn et al., 2016). Another inhibitor, guanidine, has also been shown to significantly reduce viremia and pulmonary virus titers, as well as to prevent histological lung lesions in the EV-D68 respiratory animal model (Hurst et al., 2019). Likewise, other 2C protein inhibitors, such as Jun6504 (Li et al., 2025a), R523062 (Ma et al., 2020b), and A-967079 (Tan et al., 2024), have been reported as potent and broad-spectrum antiviral agents effective against various strains of EV-D68.

IRES-mediated translation is a critical mechanism in picornavirus infection. Consequently, targeting this pathway represents a potential strategy to impede viral growth. Indeed, the antiprotozoal drug emetine has demonstrated inhibitory effects against a range of human enteroviruses, including EV-D68, at nanomolar level (Tang et al., 2020). Licochalcone A, a flavonoid compound derived from the root of Glycyrrhiza species, has also been shown to markedly inhibit EV-D68 replication via interfering with viral IRES-dependent translation (Chuang et al., 2024).

### 5.5 Antivirals targeting host factors

Beyond direct-acting antivirals, antiviral strategies may also focus on host proteins exploited by viruses to facilitate infection and replication (Hu et al., 2020; Jassey et al., 2024b). For instance, DAS181, a sialidase that cleaves α2,6-linked sialic acids on the cellular surface, has demonstrated *in vitro* potential to inhibit both the EV-D68 Fermon strain and contemporary strains via obstructing viral attachment and entry (Rhoden et al., 2015). In addition, phosphatidylinositol-4-kinase type III β (PI4KIIIβ) is actively recruited to replication sites in enterovirus-infected cells, generating a microenvironment enriched in its product, phosphatidylinositol-4-phosphate (PI4P), which in turn promotes the attraction of components essential for viral RNA at these sites (Hsu et al., 2010). Not surprisingly, enviroxime—a small molecule inhibitor of PI4KIIIβ that has progressed to clinical trials—has demonstrated efficacy in inhibiting multiple strains of EV-D68 (Sun et al., 2015; Smee et al., 2016). Similarly, oxysterol-binding protein (OSBP), a PI4P-binding cholesterol transfer protein, has been recognized as a host factor necessary for enterovirus replication. Further supporting this mechanism, protein kinase D (PKD), an enzyme involved in regulating vesicular transport through the phosphorylation and activation of its substrate PI4KIIIβ, has also been implicated in enteroviral replication (Hausser et al., 2005; Guedán et al., 2017). Importantly, specific OSBP antagonists, such as itraconazole (Spradley et al.) and OSW-1, as well as PKD inhibitors like CRT0066101, have demonstrated efficacy in inhibiting the replication of many enteroviruses (Strating et al., 2015; Guedán et al., 2017). Therefore, it is plausible to hypothesize that these OSBP and PKD inhibitors may similarly exhibit inhibitory effects on EV-D68 replication.

Unlike direct-acting antivirals, therapies targeting host factors generally reduce the probability of resistance emergence. However, since host factors often perform diverse and essential functions within host cells, antiviral agents targeting these factors may also pose significant risks if their activity is indiscriminately inhibited.

## 6 Conclusions and prospects

Over the past decade, EV-D68 has been implicated in a rising incidence of severe respiratory illnesses and permanent neurological impairments affecting thousands of children globally. Although a correlation between EV-D68 and AFM has been established, the virus has historically been neglected due to limited scientific knowledge, inadequate laboratory diagnostic capabilities, and insufficient surveillance by public health authorities. At present, the development of effective prevention and treatment strategies for EV-D68 infections constitutes an urgent public health priority. Therefore, a comprehensive understanding of the biological characteristics of EV-D68 will be imperative to facilitate the efficient advancement of vaccines, antiviral agents, and neuroprotective interventions.

Since its emergence as an endemic pathogen in 2014, significant progress has been made in elucidating the pathophysiology of EV-D68 infection, notably including the recent identification of its functional receptor, MFSD6. Nevertheless, considerable gaps remain in our understanding of EV-D68 pathogenesis. Of particular concern is the observation that, although EV-D68 has been more frequently detected in respiratory specimens than in the cerebrospinal fluid of a small proportion of AFM patients, a strong association between EV-D68 infection and AFM has been established. Despite this correlation, the underlying pathological mechanisms by which EV-D68 affects the central nervous system remain poorly understood. Moreover, similar to other RNA viruses, EV-D68 demonstrates substantial genetic diversity. Viral evolution is regarded as a main factor contributing to the acquisition of its neurovirulence properties; however, the specific driving forces responsible for this process have yet to be clearly identified. Additionally, frequent EV-D68 outbreaks underscore its potential to maintain endemic transmission within human populations. Consequently, it is imperative to persistently monitor the epidemiology of EV-D68 and to develop predictive models for future incidences and spread. Finally, given the overall genetic similarity between EV-D68 and other enteroviruses, future efforts should prioritize the identification and development of broadly cross-neutralizing antibodies. Such antibodies, which possess the capacity to neutralize a broad range of enteroviruses including EV-D68, would be of immense therapeutic and prophylactic value. Their application could provide a promising approach for combating not only EV-D68 infections but also emerging enteroviral threats.
